# Encouraging water-saving behavior during a “Moment of Change”: the efficacy of implementation intentions on water conservation during the transition to university

**DOI:** 10.3389/fpsyg.2024.1465696

**Published:** 2024-11-13

**Authors:** Kaloyan Mitev, Freya Rennison, Paul Haggar, Rebecca Hafner, Alice Lowe, Lorraine Whitmarsh

**Affiliations:** ^1^Department of Psychology, University of Bath, Bath, United Kingdom; ^2^School of Psychology, Swansea University, Swansea, United Kingdom; ^3^Climate Action Team, University of Bath, Bath, United Kingdom

**Keywords:** water use, university student, moment of change, habit discontinuity, implementation intention

## Abstract

Water saving behavior is of substantial importance in climate change mitigation and resilience, including reducing time spent in the shower. However, water use is, for many, a strong habit, and, as such, incorporating new water saving behaviors into one's domestic routines may be unsuccessful. In this study, we consider the extent to which a composite behavior change intervention (of water-saving information, implementation intention formation, and monitoring using a shower timer) is effective in reducing the domestic water consumption of new university students who have recently moved into university accommodation. We focus on aspects of the habit discontinuity hypothesis, namely that a natural moment of change facilitates behavior change by weaking existing habits. The intervention was found to be effective, increasing the frequency of self-reported water-saving behavior over behavior measured in a control group. However, shower times, and water usage (measured at the residential level), were not affected by the intervention, and strength of existing habits, readiness to change water behavior, and recency of starting university were each not significantly associated with the effectiveness of the intervention. However, all participants (irrespective of intervention) increased water-saving behavior and reduced shower time during the study, with residential water usage being less for residences with more participating students. Contrary to expectations, the timing of the intervention did not show a clear effect upon the efficacy of the intervention. We discuss these findings with respect to moments of change and habit discontinuity theory as well as implications for practical behavior change interventions.

## Introduction

The management and treatment of freshwater accounts for a large proportion of global energy consumption (IEA, 2017) and produces methane and nitrous oxide emissions (Crippa et al., 2020). Demand for freshwater is increasing due to urbanization and development, while water availability is declining due to climate change (He et al., 2021; Finley and Basu, 2020). For example, in England, the Environment Agency predicts that the country will be short of 3,435 million liters per day by 2050 (Environment Agency, 2020). Therefore, there is an imperative to reduce water consumption, both to mitigate, and to adapt to, climate change. Reducing household demand for water is an essential part of reducing overall water usage (Environment Agency, 2020) and the UK government has identified supporting households to reduce their water use as a key element of managing the UK's water supply (DEFRA, 2023).

Despite this urgency, research focused on changing behavior around water use has found mixed results. For instance, interventions focusing on providing information about water usage are not consistently effective in reducing water consumption (Grilli and Curtis, 2021) and water usage is insensitive to changes in price, particularly when money is not a priority (Garcia-Valiñas et al., 2014; Goette et al., 2019; Tijs et al., 2017). One possible explanation is the role of habits, which are a relatively good predictor of water usage (Garcia-Valiñas et al., 2014; Gregory and Leo, 2003). A habit is an automatic association between a contextual cue and a response (e.g., Rebar et al., 2018; Verplanken and Aarts, 1999), which is formed through repetition and is context dependent (Gardner and Lally, 2018). Usually, habitual behavior is carried out via an unconscious association between cue and action (Verplanken and Aarts, 1999) and this property of automaticity makes many environmental behaviors difficult to change or cultivate. Without conscious deliberation, people's good intentions may not match their automatic, less deliberative habits (Gardner, 2009; Fujii and Garling, 2005).

Consequently, changing the environments which trigger habits might provide a way toward behavior change (Carden and Wood, 2018). When a person's context changes, they are no longer exposed to the contextual cues that automatically trigger their behaviors. This disruption in context might create a window of opportunity, during which a person's behaviors are more conscious, and therefore more susceptible to behavioral interventions (Verplanken et al., 2018). This idea is known as the “Habit Discontinuity Hypothesis” (Verplanken et al., 2018).

In fact, there are certain periods in people's lives which represent natural changes to people's physical and/or social environments which are known as *Moments of Change* (MoCs) and could constitute instances of habit discontinuity (Verplanken et al., 2018). Some examples include residential relocation, becoming a parent for the first time, and work retirement. In addition, research suggests that behavioral interventions might be more effective when applied during MoCs (Verplanken and Whitmarsh, 2021). For example, transport interventions have been found to be more effective when participants have recently moved house and/or job (Thøgersen, 2012; Ralph and Brown, 2019), while Maréchal (2010) found that residents who had recently moved house were more likely to apply for energy subsidies. Considering water consumption and MoCs, one study found the average length of showers decreased after the disruption of the Covid pandemic (Swaffield et al., 2023), while another reported that participants who had moved house more recently were more likely to maintain new water-saving habits—suggesting that contextual change enables habit change (Dean et al., 2021). Taken together, these findings suggest targeting water consumption during MoCs may be an effective way of influencing behavior change.

An understudied but important MoC is the start of university. In 2023, 35.8% of 18-year-olds in the UK started an undergraduate degree (Bolton, 2024). This makes the start of university one of the most widespread MoCs that young people go through during early adulthood. In addition to starting a course of study, the start of university often also entails changes in both the physical and social environments and students residentially relocate to live on or near the university campus. Furthermore, most incoming students in the UK are between 16 and 18 years of age, starting university after completing their secondary education. For these students, starting university coincides with an important developmental period, i.e., late adolescence, defined by a focus on self-exploration (Arnett, 2016), identity formation (Erikson, 1968), and increasing personal independence (Drake et al., 2016). This period is characterized by resistance to parental persuasion, increased self-determination, nonconformity, an internal locus of control, and instrumentality (Beyers et al., 2003). As such, for many of these students, the start of university may also be an important moment for experimenting with new behaviors and lifestyles that reflect personal identity, some of which will stick with them in the long-term. Finally, interventions involving participants in their late adolescence have demonstrated success in altering behavioral practices over the long term (Borman et al., 2018; Hayes et al., 2019). This suggests that conducting interventions with young people going through a MoC, such as starting university, might provide an important pathway to pro-environmental behavior change including water use. This is the focus of the present paper.

However, there is no consensus on how close in time to a MoC an intervention must be introduced in order for it to be most effective. For example, Verplanken and Roy (2016) tested an intervention to foster a range of pro-environmental behaviors and found that it was more effective for participants who had recently relocated, but only if they had relocated in the last 3 months. This suggests that the first 3 months following a MoC might offer a ‘window of opportunity' critical for a successful behavioral intervention (cf. Lally et al., 2010). Similarly, Schäfer et al. (2012) suggest that 6 months after a MoC might be too late for an intervention to capitalize on habit discontinuity. Thomas et al. (2016), by contrast, found residential relocation increased the probability of attitude-led car-use reduction within a few months of the event, but also found evidence for some reduction up to 12 or even 24 months after the event. Therefore, this research also tests the idea of a window of opportunity for applying interventions during MoCs by conducting the intervention twice—first with students who had just started university in the previous 1–2 months, and then with students who had started university 5–6 months prior to taking part.

While implementing behavioral interventions within a MoC might enhance their efficacy, the intervention approach also plays a crucial role in maximizing potential outcomes. One commonly used method to promote pro-environmental behaviors (PEBs) involves providing information regarding the consequences of climate change and the significance of engaging in PEBs. However, solely relying on an informational campaign alone typically yields less effective results than when information is integrated with other strategies in a composite intervention, particularly for changing habitual behavior (Whitmarsh et al., 2021). Therefore, in the present research, we combined an informational campaign with a habit-breaking intervention (implementation intentions) and performance feedback (providing participants with a shower timer).

*Implementation intentions* is a method of changing behavior that can be especially effective in breaking habits (Gollwitzer and Oettingen, 2020). This method involves a person creating an “if-then” plan in which they associate a contextual cue (e.g., an event or circumstance) with a new behavior (e.g., if I leave the kitchen, then I will turn the light off). This new contingency is formed to replace the existing, undesired, habit with a deliberate alternative action, the plan likely also increasing one's awareness of the existing environmental cues (Aarts et al., 1999). Such interventions have been successful in a range of domains, including changing recycling and transport behaviors (Holland et al., 2006; Rise et al., 2003; Bamberg, 2000). Although setting targets for water consumption has had some success in a previous study (Walton and Hume, 2011), to the best of our knowledge, implementation intentions specifically have not been tested on water-saving behaviors.

Providing performance feedback about goal-focused tasks can help people meet these goals because knowing whether their current efforts are successful allows them to alter or maintain their approach accordingly in the pursuit of continuing success (Locke and Latham, 2002). Hence, providing this information regarding task performance has often been used as an aspect of goal-directed interventions (Locke and Latham, 2002; Osbaldiston and Schott, 2012; Haggar et al., 2023). In our research, a shower timer was provided to the experimental group so participants can use shower-time information to improve their performance of this behavior, which has a substantial impact upon domestic water-use (Waterwise, 2024).

In addition to the attributes of the MoC and the intervention strategy, there are likely to be individual factors which enhance or inhibit behavior change. Habit strength, particularly the dimension of automaticity (action without conscious deliberation), is associated with the maintenance of behavior (Verplanken and Orbell, 2003; Gardner et al., 2012). Hence, we would expect the intervention strategy might be more effective for those participants with weaker existing habits and for those participants experiencing a MoC, for whom stronger habits may be temporarily weaker (Verplanken et al., 2018) but less effective for others with strong habits, who may habitually neglect the information (Verplanken and Aarts, 1999) or otherwise follow established routines automatically. Readiness to change may also affect intervention efficacy: while some people may have been considering water-reductions for some time, the idea of reducing personal water-use may be quite novel to others. In the transtheoretical model of behavior change, people who successfully change their behavior are described as moving through five discrete stages of change, from giving the idea little consideration (precontemplation) to thinking about it, planning how to execute a change, putting the change into effect, and then maintaining the change for an extended period (Prochaska and DiClemente, 1983). Research shows that those further along in these five-stages, and hence more “ready to change”, are also more receptive to implementation intention interventions (Bell et al., 2016; Armitage and Arden, 2008), implying that the success of our composite intervention may depend upon existing readiness to change, with those in the earliest stage potentially entirely unreceptive. More broadly, in predicting or explaining the failure of behavior changes within intervention studies in the field, other inhibiting factors upon change exist, particularly more embedded issues such as the design of the material water system, with its emphasis upon convenience over conservation, and existing social norms and culture expectations around water-use and cleanliness (Hand et al., 2005).

In this article, we report the results of a study of students starting their first year of university to consider how a composite intervention may be enhanced during this MoC, assessing behavior change across a three-week period. We compare a composite intervention (comprising an implementation intention task, water conservation information, and shower timer) to a control condition. In a MoC exposure group we conducted the intervention 1–2 months after the start of their first year of university, while in the non-MoC group we conducted the intervention ~5–6 months after the start of their first year of university. We evaluated four hypotheses.

**Hypothesis 1**: In line with previous research (Holland et al., 2006; Rise et al., 2003; Bamberg, 2000; Walton and Hume, 2011), we anticipate that the composite intervention will lead to positive changes in water consumption (reduced shower time, increased water-saving behaviors) compared to the control condition.

**Hypothesis 2**: In line with previous research (e.g., Bell et al., 2016; Armitage and Arden, 2008) we anticipate that the intervention will be more effective for participants who have higher readiness to change concerning saving water.

**Hypothesis 3**: Consistent with Verplanken and Orbell (2003), we anticipate that the intervention will be more effective for participants whose water behavior is less habitual.

**Hypothesis 4**: In line with previous research on the Habit Discontinuity Hypothesis (Verplanken and Roy, 2016), we expect the intervention to be more efficacious for those students who had started university more recently (1–2 months before) than for those who had started university less recently (5 to 6 months before).

## Methods

### Design and participants

We used a 2 × 2 × 3 mixed-factorial design: 2 (*Intervention*: intervention or control) × 2 (*MoC*: 1–2 months or 5–6 months) × 3 (*Time*: baseline, 7-days and 21-days). Student participants were sampled from university campus residential accommodation (residences) on a purpose-built university campus in a suburb of a city in the United Kingdom. The intervention was administered at baseline. Two dependent variables (*shower time* and *water-saving behavior*) and two further independent variables (*readiness to change* and *habit-strength*) were measured by questionnaire; a third dependent variable, (residential) *water usage* was recorded across a two-month study period.

Assignment to intervention/control conditions was randomized at the residential level, across 55 different buildings: 45 small buildings (each small building housing ~11 to 13 students) and 10 large buildings (each large building housing between 34 and 303 students; large building M = 133.2, SD = 70.53). The intervention was assigned to 20 small buildings and 6 large buildings. MoC groups (1–2 months and 5–6 months) were recruited in separate waves to ensure that all participants were from the same student cohort. The first wave (1–2 months group) was recruited from the small residences and the second wave (5–6-months group) was recruited in the large residences; thus the two samples were independent.^1^ During recruitment, participants were informed that the study aimed to explore the impacts of the cost of living on lifestyle. Our initial sample across both waves consisted of 186 first year university student participants: age range 18–22 (M = 18.7); 48.4% female; 47.3% male; 1.6% non-binary/third gender; 2.7% preferred not to say. The questionnaire on day-7 was completed by 103 participants (55.4%) and the questionnaire on day-21 by 94 participants (50.5%).

### Materials, measures and variables

#### Intervention

The composite intervention consisted of: (1) water-saving information; (2) implementation intention task; (3) a shower timer and instructions. Information concerned water-scarcity and the importance of individual water-use reduction, including eight behaviors (hints/tips) for water-saving. Behaviors were drawn from content analysis of current water-saving in the top six websites found from Google search-engine results (searching “Water saving hints and tips UK”). The implementation intention task (Bell et al., 2016) asked participants to reflect on the eight behaviors they had read, then participants were asked to select four of these behaviors and formulate “if/then” statements for these using a pro-forma table. Several examples were given for guidance. Finally, instructions were given concerning the shower timer. This encouraged them to reduce shower time to under 4 min, and provided brief operating instructions: the shower timer was a sand-timer intended for use in the shower which had a duration of ~4-min.

#### Filler-task

Participants in the control condition did not receive the composite intervention. Instead, they completed a reading comprehension filler-task (e.g., Mometrix, 2023). Participants were asked to read a paragraph of rules for a word-game, and it was stated that they may be asked to play this game with other participants later in the study. There followed four multiple-choice questions about the details in the text. The task's subject matter was a geographical word game and so was not related to water-saving or to sustainability.

#### Shower time

A single open-answer question read: “How long, on average do you spend in the shower? Please write your answer in minutes in the box below. This can be a rough guess”. We excluded three outlying participants from shower time analyses.^2^

#### Water-saving behaviors

These were measured with an eight-item self-report scale, adapted from previous research on energy-saving behavior by Hafner et al. (2020). Water-saving behaviors were identified in the same content analysis used to produce the informational component of the intervention. We asked the question “thinking about the last 2 weeks, how often have you taken the following actions”, with five answer options [“never” (1), “very rarely” (2), “sometimes” (3), “often” (4) and “always” (5)] and eight items related to different water-saving behaviors. Items included, for example, “When boiling the kettle I have only filled it with as much water as a I need” and “When washing fruit and vegetables I have used a bowl of cold water rather than continuously running the tap”. Seven of eight items were scaled using a mean average across all item scores (Cronbach's alpha = 0.66); one (tooth brushing) was excluded as it consistently reduced reliability across all three time points. To reduce the salience of water-use and so discourage demand effects (Orne, 1962), these items were randomized within a block of 16 items, with the other eight items reflecting unrelated lifestyle behaviors, such as “I have exercised or played sports” and “I have spent time in voluntary work, e.g., befriending or answering a help-line” (e.g., Haggar et al., 2023). The Supplementary material contains the complete questionnaire, including all 16 items.

#### Habit strength

Participants were asked to rate their agreement with each of four statements, on a five-point scale from “strongly disagree” (1) to “strongly agree” (5). The four statements were those from the Self-Report Behavioral Automaticity Index (SRBAI: Gardner et al., 2012), the purpose of which is to measure the automaticity of a behavior; automaticity is a necessary condition of habit, and stronger habits are more automatic (Verplanken and Orbell, 2003). Each statement reflected domestic water use, beginning with the phrase “using water in my home is something I do…”, e.g., “using water in my home is something I do automatically” and “using water in my home is something I do without having to consciously remember”. Scores for the four statements were scaled using a mean average across all item scores (Cronbach's alpha = 0.80), however this variable had marked left skew (Median = 4), so, for analysis, we re-categorized scores into a two-level factor: “strong” (>4; *n* = 83) or “weak” (≤ 4; *n* = 102).

#### Readiness to change

This was measured using a single question with an ordinal scale of five response options, adapted from Bell et al. (2016). For analysis, due to small and uneven sub-sample sizes, we re-categorized scores into three ordinal categories: *low* (“I currently do not try to save water and I am not thinking about starting” (1) or “I currently do not try to save water but I am thinking about starting” (2); *n* = 55), *medium* (“I currently try to save water but not on a regular basis” (3); *n* = 83), or *high* (“I currently try to save water but have only done so recently/in the last 6 months” (4) or “I currently try to save water and I have done so for a long time/longer than 6 months” (5); *n* = 47).

#### Demographics

The participants completed questions about their age, gender, and place of residence (in the baseline questionnaire only). These and all other questionnaire items are included in the Supplementary material.

#### Water meter variables

Water meter readings for the study period were made available to us and were in cubic meters (m^3^). Data was not available for all buildings but was available at 24 small buildings (12 assigned to the intervention) and 8 large buildings (5 assigned to the intervention). To facilitate comparability between different buildings, and better allow for possible confounding between the MoC groups and building sizes, small building readings were aggregated into one of two data-points: small buildings in the intervention condition and small buildings in the control condition. The maximum number of residents was derived from existing records (Universities UK, 2024) and used as an explanatory variable. We reasoned that the impact of the intervention upon this water usage would be negatively moderated by the number (or proportion) of study participants in a residence (i.e., varying exposure to the intervention), hence we also included the percentage of participants in a residence (based on maximum occupancy) and the interaction (product) variable for this percentage and participating in a building whose participants received the intervention as explanatory variables.

### Procedure

We received ethical approval from the Ethics Committee at the University's Department of Psychology (Reference number: 23-016) before launching the study. University student volunteers from the “climate champions” programme implemented initial recruitment in-person, visiting student residences on campus to this purpose. Those recruited in residences assigned to the intervention condition were given a shower-timer and asked to scan a QR-code that hyperlinked to an online questionnaire in which the intervention was embedded. Those recruited in control residences were not given a shower-timer and were asked to scan a different QR-code that hyperlinked to an online questionnaire identical to that used in the intervention condition, except that a filler-task was substituted for the intervention. The questionnaire began with a briefing page concerning the study and the terms of participation and data retention. Here, it was stated that: “the project aims to explore engagement with different lifestyle behaviors and impacts of the cost of living”. Participants then gave their informed consent, completed either the intervention material or the filler task, and then answered questionnaire questions. Follow-up questionnaires were sent to participants 7-days later and 21-days later. These were identical, except that the 21-days questionnaire ended with a written debrief covering the aims and purpose of the study and thanking participants for their participation. All participants were remunerated and received additional remuneration (prize draw entry) if they had completed all three questionnaires. Recruitment took place in two waves. The procedure was identical in each wave. Participants in the 1–2-month group (first wave) were recruited from October to December 2023 and water meter readings were taken before the study, in October, and on 13th November.^3^ The second wave (when the student cohort had been resident at the university for ~5–6 months) were recruited from February to April 2024, and water meter readings were taken in February and April.

### Data analysis plan

Data was cleaned, including the removal of outliers (defined as greater than the third quartile plus three times the interquartile range or less than the first quartile minus three times the interquartile range). Analyses were conducted using SPSS version 29. Hypothesis tests using survey data were made using mixed Analysis of Variance (ANOVA) models. To conserve statistical power, given our relatively small sample size, several models tested fewer factors and/or fewer levels than described in our study design. All dependent variable measures deviated from the normal distribution. Hence, aligned rank transformed (ART) data was used through the ARTool software (Wobbrock et al., 2011; Elkin et al., 2021).^4^ As a repeated-measures assumption of sphericity was rarely met, the Greenhouse-Geisser correction was used (Blanca et al., 2023). Planned comparisons were t tests on ART data with Bonferroni correction applied for multiple comparisons. An analysis of water usage at the residential level was made using multiple linear regression.

## Results

### Baseline descriptive statistics

Descriptive statistics for measured variables (Table 1) show mean averages for water-saving behavior and readiness to change to be close to the scale mid-point (3), whereas the mean average for water-saving habit strength is somewhat greater than the scale mid-point. Skewness and kurtosis statistics indicate (on a Z ± 3.29 criterion) that: (a) age and shower time are both right-skewed and leptokurtic; (b) habit strength is left-skewed (Mishra et al., 2019). Descriptively, intervention participants report somewhat shorter showers, M(SD) = 9.0 (5.35) than control participants, M(SD) = 10.3 (5.84) and somewhat more water-saving behavior, M(SD) = 2.7 (0.74), than control participants, M(SD) = 2.5 (0.76), at baseline. Mann-Whiney U-Tests were made to assess these differences, as well as to compare habit strength and readiness to change between groups at baseline, however differences were not statistically significant, either for shower-time, U(Z) = 3782.5 (1.37), *p* =0.171, water-saving behavior, U(Z) = 3,619.0 (1.81), *p* = 0.071, habit strength, U(Z) = 3,755.0 (1.66), *p* = 0.097, or readiness to change, U(Z) = 4,272.5 (0.01), *p* = 0.994.

**Table 1:** Baseline descriptive statistics.

	***N***	**Minimum**	**Maximum**	**M (SD)**	**Skewness (SE)**	**Kurtosis (SE)**
Age	186	18	22	18.71 (0.826)	1.34 (0.178)	2.53 (0.355)
Shower time	185	2	45	9.95 (6.031)	2.14 (0.179)	7.23 (0.355)
Water-saving behavior	185	1	5	2.56 (0.753)	0.46 (0.179)	−0.01 (0.355)
Habit strength	185	1.75	5	4.04 (0.749)	−0.67 (0.179)	0.10 (0.355)
Readiness to change	185	1	5	3.01 (1.163)	0.18 (0.179)	−0.49 (0.355)

### Intervention efficacy

#### Shower time

**Figure 1:**
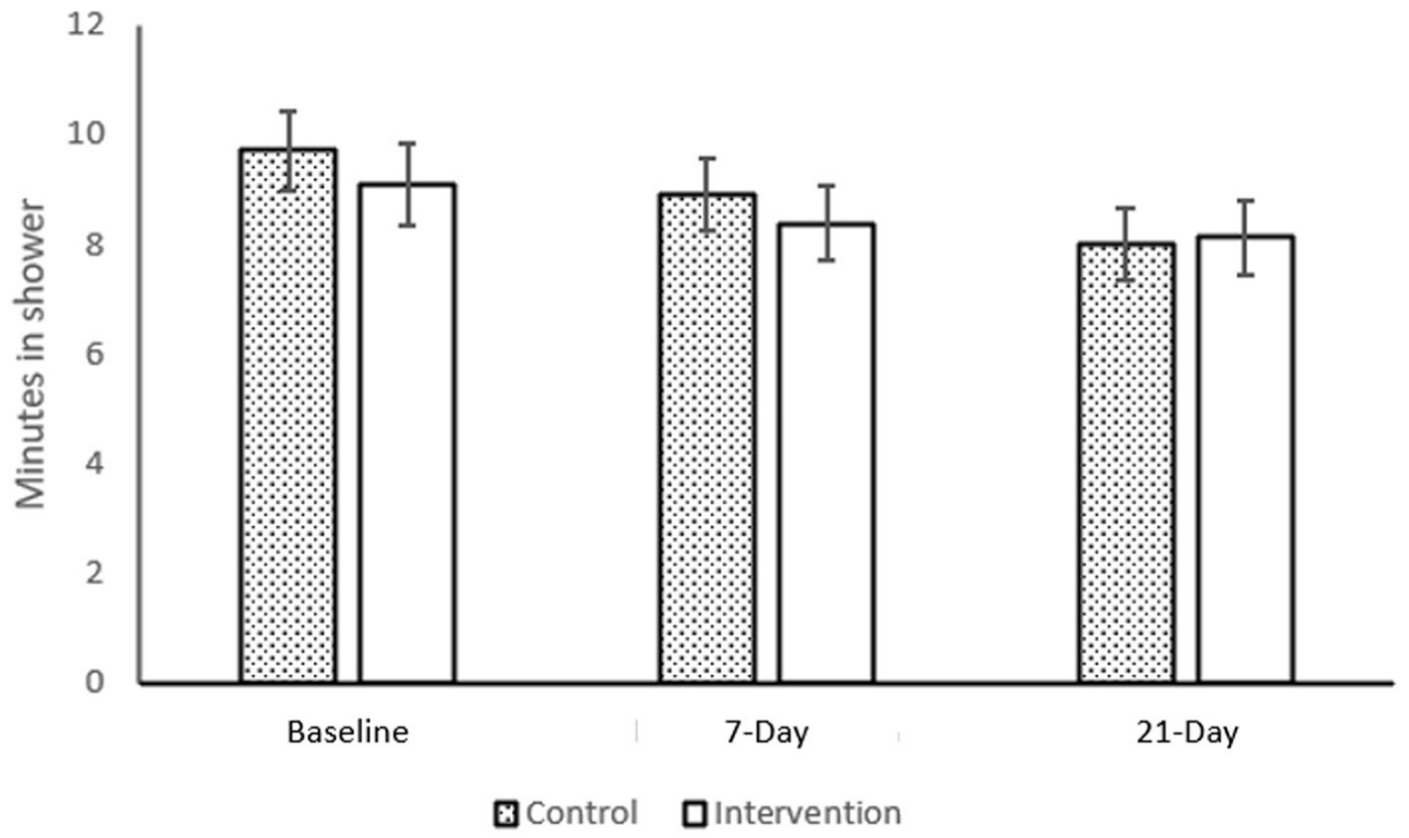
Intervention and shower time. Control *n* = 47, Intervention *N* = 43. Error bars show 1SE.

A 2 (*Intervention*: Intervention or Control) × 3 (*Time:* Baseline, 7-day, and 21-day) mixed ANOVA showed a significant main effect of *time*, *F*_(1.58, 139.38)_ = 15.50, *p* < 0.001, η^2^ = 0.150, but the main effect of intervention and the interaction effect were not statistically significant. Descriptive comparison (Figure 1) showed shower time to be descriptively longer at baseline, M(SD) = 9.43 (4.99), than at either 7-days, M(SD) = 8.66 (4.41), or 21-days, M(SD) = 8.07 (4.43).

#### Water-saving behavior

**Figure 2:**
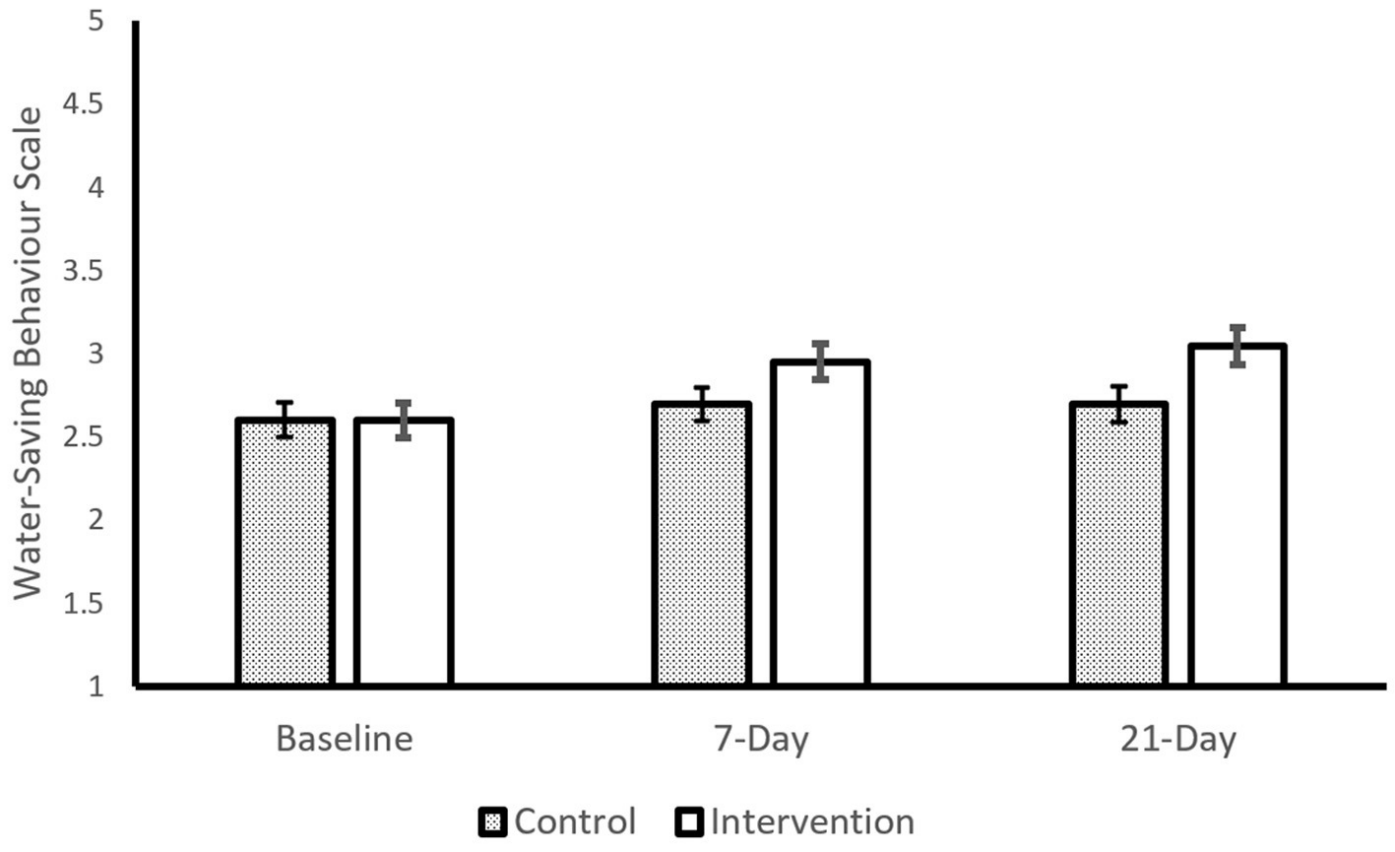
Intervention and water-saving behavior. Control *n* = 49, Intervention *N* = 44. Error bars show 1SE. Water saving behavior scales ranges between 1 and 5.

A 2 (*Intervention*: Intervention or Control) × 3 (*Time*: Baseline, 7-day, and 21-day) mixed ANOVA showed a significant main effect of *time*, *F*_(1.805, 164.23)_ = 12.63, *p* < 0.001, η^2^ = 0.122, and a significant *interaction* effect, *F*_(1.83, 166.05)_ = 4.35, *p* = 0.017, η^2^ = 0.046, but the main effect of intervention was not statistically significant, *F*_(1, 91)_ = 1.86, *p* = 0.176. Descriptive comparison (Figure 2) suggested that baseline water-saving was less, M(SD) = 2.60 (0.706), than either 7-day water-saving, M(SD) = 2.82 (0.729), or 21-day water-saving, M(SD) = 2.86 (0.760). Planned contrasts showed that intervention participants increased water-saving behavior between baseline and day-7, M = +0.35, t_(43)_ = 3.09, *p* < 0.01, and between baseline and day-21, M = + 0.45, t_(43)_ = 4.16, *p* < 0.001, but the water-saving of control participants did not show statistically significant changes.

#### Water usage

On average, the 10 residences used 1,000.7 m^3^ of water (SD = 655.18 m^3^) over the test periods and housed 166.6 residents (SD = 72.89), ranging between 99 and 303 residents, of whom between 10 and 33, M(SD) = 19.0 (8.88), participated in the study in each residence. Table 2 shows the results of a multiple linear regression of residential water usage on maximum number of residents, the number of study participants as a percentage of the maximum number of residents, and whether participating residents received the composite intervention (1) or not (0). The model fit was high, R^2^ adj. = 0.955, likely due to close correlation between maximum residency and water usage, r = 0.957, *p* < 0.001. While the intervention effect was not statistically significant, the proportion of residents participating showed a statistically significant negative association with water usage, indicating that the more people participated in the study the less water was used during the study period, irrespective of intervention or control conditions.

**Table 2:** Residential water usage regressed on residents, experimental condition and estimated percentage of residents participating.

					**95% CI**	
**Variable**	**B (SE)**	**Beta**	**t**	***p***	**Low**	**High**
Intercept	81.18 (209.193)		0.39	0.71	−430.7	593.1
Residents	7.56 (0.707)	0.84	10.69	< 0.001	5.83	9.29
Experiment	77.41 (89.731)	0.06	0.86	0.42	−142.2	297.0
%Participants	−31.38 (9.645)	−0.26	−3.25	0.02	−54.98	−7.78

N = 10. “Residents” is maximum number of residents, i.e., bedrooms. “Experiment” is participation in intervention (1) or non-intervention (0) condition. “%Participants” is the number of participants in the residence divided into Residents. R^2^ adj. = 0.955. Analysis expressing “%Participants” as a count variable yields similar (i.e., statistically significant) results: B(SE) = −17.05 (5.624), p < 0.05, 95%CI [-30.8, −3.29]. Entering an Experiment^*^%Participants interaction (product) variable neither explains additional variance nor reaches statistical significance as an explanatory variable. Explanatory variables are not statistically significantly correlated: Residents with %Participants (r = −0.44, p = 0.204), Residents with Experiment (r = 0.05, p = 0.894), or %Participants with Experiment (r = 0.048, p = 0.894).

#### Summary

Hypothesis 1 stated that “the composite intervention will lead to positive changes in water consumption (reduced shower time, increased water-saving behaviors) compared to the control condition”. We found evidence that the intervention led to increases in water-saving behaviors, but we did not find evidence that the intervention led to reduced shower time or lower residential water usage. However, overall, we found evidence that positive changes in water consumption did occur across time: shower times reduced, water-saving behavior increased, and residential water-usage was smaller when more study-participants were in residence.

### Readiness to change and existing habits

#### Shower time

A 3 (*Readiness to change*: low, medium or high) × 3 (*Time*: Baseline, 7-day and 21-day) mixed ANOVA using the intervention group subset only (n = 43) showed a significant main effect for *time*, *F*_(1.44, 57.49)_ = 4.79, *p* = 0.021, η^2^ = 0.107, but no significant main effect of readiness to change or interaction effect. A 2 (*Habit Strength*: low or high) × 3 (*Time*: Baseline, 7-day and 21-day) mixed ANOVA using the intervention group subset only (n = 43) showed a significant main effect of *time*, *F*_(1.48, 60.67)_ = 4.32, *p* = 0.027, η^2^ = 0.095, but no significant main effect of habit strength or a significant interaction.

#### Water-saving behavior

A 3 (*Readiness to change*: low, medium or high) × 3 (*Time*: Baseline, 7-day and 21-day) mixed ANOVA using the intervention group subset only (n = 44) showed significant main effects of *time*, *F*_(1.55, 63.54)_ = 12.74, *p* < 0.001, η^2^ = 0.237 and of *readiness to change*, *F*_(2, 41)_ = 4.39, *p* = 0.019, η^2^ = 0.176. However, the interaction effect was not statistically significant. Descriptively, water-saving behavior was greater with greater readiness of change, from a mean average score of 2.58 (low) to 2.87 (medium) and to 3.38 (high). Planned comparisons for behavior changes following baseline within each level of readiness to change showed only one statistically significant difference (after Bonferroni correction): those with low readiness to change showed greater water-saving behavior after 21 days, M = +0.56, t(14) = 3.10, *p* = 0.004. A 2 (*Habit Strength*: low or high) × 3 (*Time*: Baseline, 7-day and 21-day) mixed ANOVA using the intervention group subset only (n = 44) showed a significant main effect of *time*, *F*_(1.56, 64.14)_ = 13.79, *p* < 0.001, η^2^ = 0.252, however neither the main effect of habit strength nor the interaction effect was statistically significant.

#### Summary

Hypothesis 2 stated that “In line with previous research (e.g., Bell et al., 2016; Armitage and Arden, 2008) we anticipate that the intervention will be more effective for participants who have higher readiness to change concerning saving water”. We found no evidence that readiness to change affected changes in water-saving behavior or shower time in the intervention group. Hypothesis 3 stated that: “Consistent with Verplanken and Orbell (2003), we anticipate that the intervention will be more effective for participants whose water behavior is less habitual”. We found no evidence that habit strength affected changes in water-saving behavior or shower time in the intervention group. Hence, we found no evidence to support either Hypothesis 2 or Hypothesis 3.

### Starting university as a moment of change

#### Shower time

**Figure 3:**
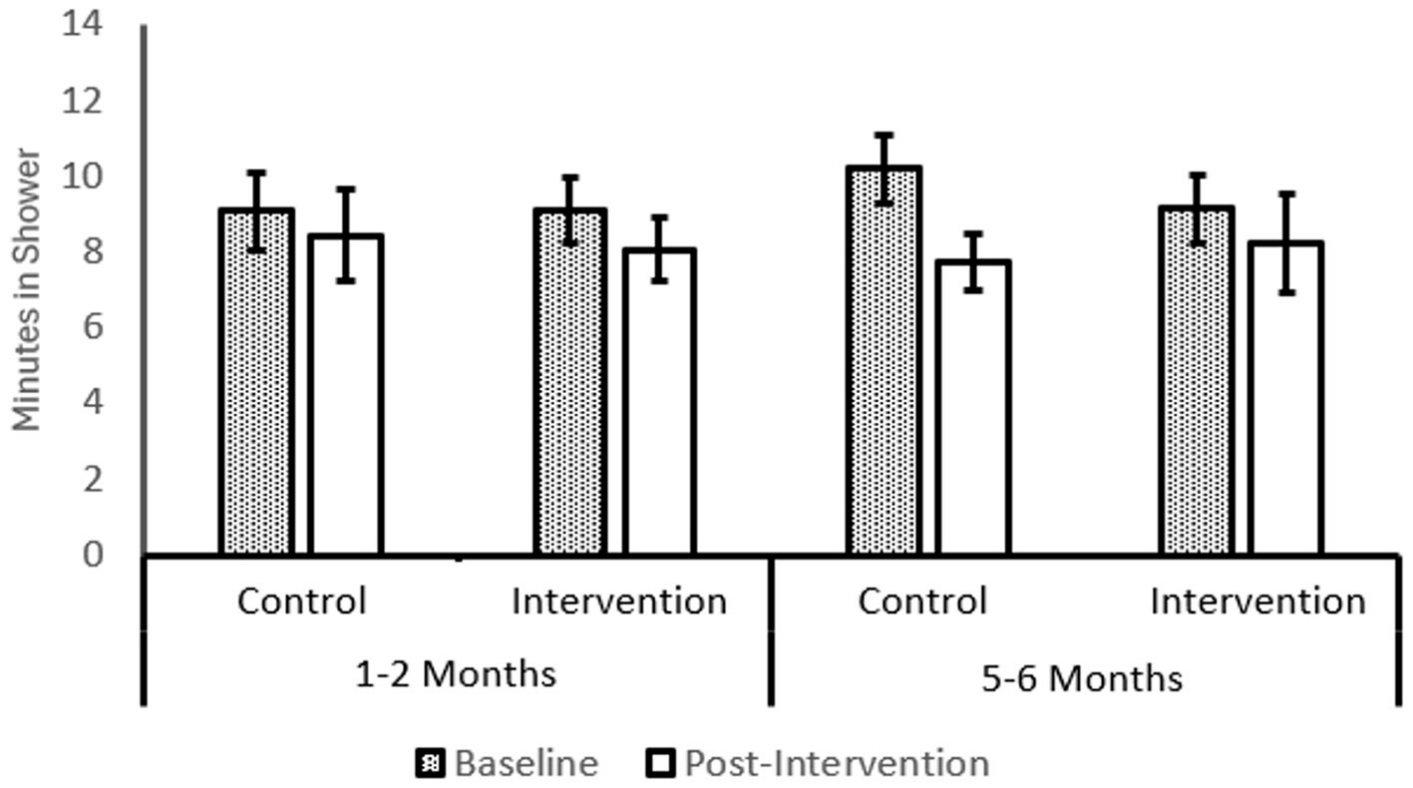
Shower Time with intervention and moment of change. Post-intervention period is 21-days. MoC is 1–2 months after starting university. Non-MoC is 5–6 months after starting university. MoC control *N* = 20; MoC Experimental *N* = 29; Non-MoC control *N* = 27; Non-MoC experimental *N* = 14. Error bars show 1SE.

A 2 (*Intervention*: Intervention or Control) × 2 (*MoC*: 1–2 months or 5–6 months) × 2 (*Time*: Baseline and 21-day) mixed ANOVA showed a significant main effect of *time*, *F*_(1, 86)_ = 25.06, *p* < 0.001, η^2^ = 0.226 and a significant *time* × *MoC interaction* effect, *F*_(1, 86)_ = 8.35, *p* = 0.005, η^2^ = 0.088, however, other effects, including the three-way interaction effect, were not statistically significant. The pattern of change is described in Figure 3. Planned comparisons showed that with the intervention shower time reduced by around two and a half minutes over 21 days in the 5–6-month group, M = −2.48, t_(28)_ = 3.06, *p* < 0.01, and without the intervention shower time reduced by around 1 min over 21 days in the 1–2-month group, t_(27)_ = 2.49, *p* < 0.01. The observed reductions in the 5–6-month group reduced average shower time from a higher level (of around 10 min) to times more comparable to the 1–2-month group (of around 8 min).

#### Water-saving behavior

**Figure 4:**
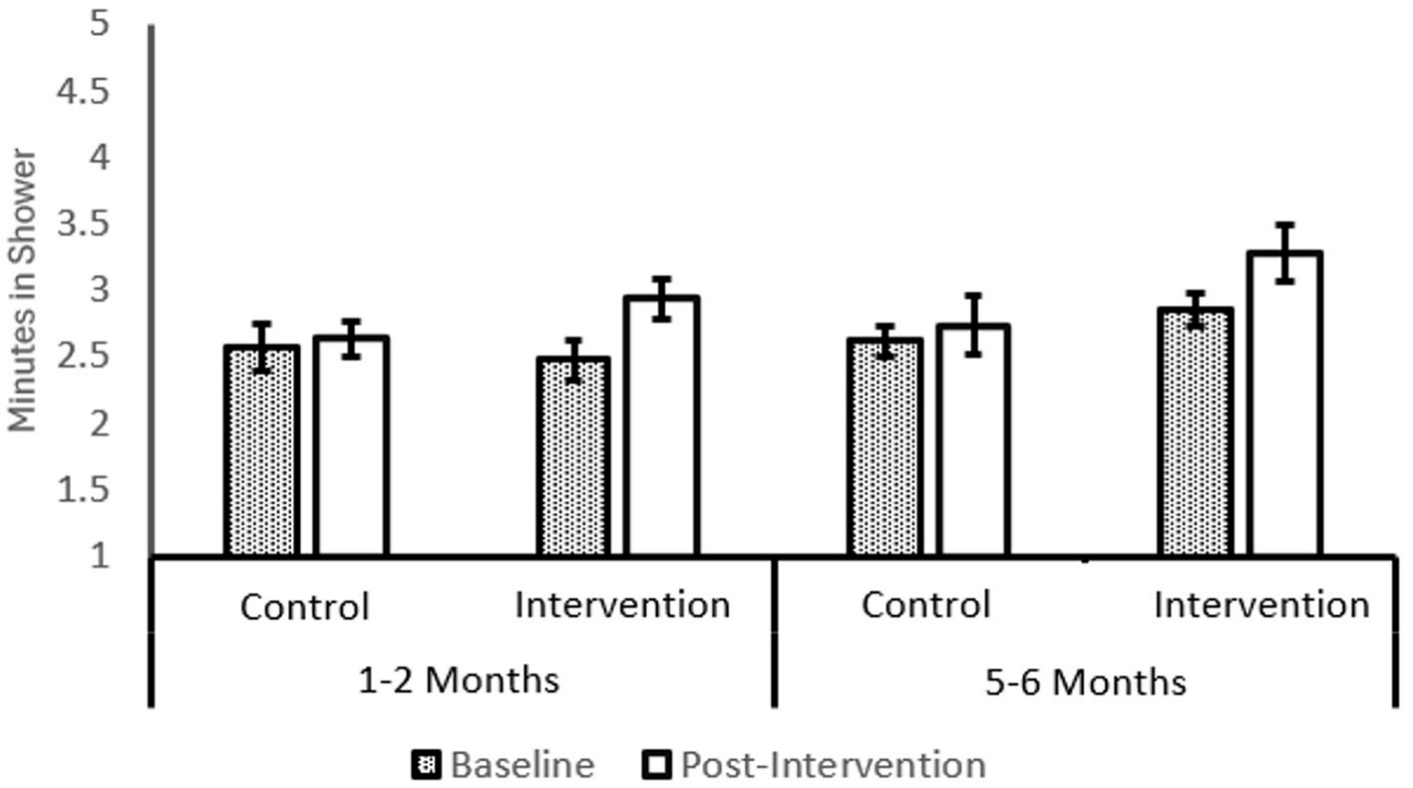
Water Saving Behavior with Intervention and Moment of Change. Post-Intervention period is 21-days. MoC control *N* = 20; MoC Experimental *N* = 29; Non-MoC control *N* = 30; Non-MoC experimental N=14. Error bars show 1SE. Water Saving Behavior Scales ranges between 1 and 5.

A 2 (*Intervention*: Intervention or Control) × 2 (*MoC*: 1–2 months or 5–6 months) × 2 (*Time*: Baseline and 21-day) mixed ANOVA showed a statistically significant main effect of *time*, *F*_(1, 89)_ = 15.32, *p* < 0.001, η^2^ =0.147, and a significant time-intervention interaction, *F*_(1, 89)_ = 6.25, *p* = 0.014, η^2^= 0.066. However, other effects, including the 3-way interaction effect, were not statistically significant. Descriptive patterns are shown in Figure 4.

#### Summary

Hypothesis 4 stated that “the intervention would be more efficacious for those students who had started university 1–2 months previously than for those who had started 5–6 months previously”. We found no clear evidence that the intervention was more efficacious in the 1–2-month group than in the 5–6-month group. For water-saving behavior, we found no evidence of any differences relating to MoC. For shower time, we found no evidence that the intervention was more effective in either MoC condition, but our results showed that the greatest reductions occurred in the 1–2-month group that did not receive the intervention and the 5–6-month group that did.

### Overall summary

Our analysis supports the following findings. (1) Across 21-days, both outcome variables improved: water-saving behavior increased, shower-time decreased. (2) Residential water-use across 21-days was negatively associated with the number of study participants living in the residence. (3) The intervention increased water-saving behavior. (4) This increase was *not* affected by levels of either readiness to change or habit strength. (5) The intervention did *not* reduce shower time. (6) No intervention effect on shower time was evident at different levels of either readiness to change or habit strength. (7) At all three timepoints, those with greater readiness to change engaged in more water-saving behavior. (8) Across 21-days, the effect of the intervention upon water-saving behavior and shower time were the same for those who experienced a MoC (the 1–2-month group) and for those who did not (the 5–6-month group). (9) However, irrespective of the intervention, the non-MoC (5–6-month) group showed greater reductions in shower time across 21-days than did the MoC (1–2-month) group.

## Discussion

Water conservation is a key element in mitigating the effects of global climate change. While demand for water increases, its supply becomes increasingly difficult, even in temperate regions (Environment Agency, 2020). A key response is to reduce demand, particularly through domestic water practices, such as taking shorter showers and conserving water (DEFRA, 2023). However, water-use behaviors can become ingrained habits (Garcia-Valiñas et al., 2014; Gregory and Leo, 2003), and as such difficult to change (Verplanken and Aarts, 1999). This article reports the results of a longitudinal survey-based experiment the aim of which was to study the water use behavior of new university students, on the premise that this is an optimal moment to affect behavioral change (Borman et al., 2018; Hayes et al., 2019).

Moments of Change (MoCs), such as moving house and beginning university, have been hypothesized to be ‘windows of opportunity' during which behavior changes are more likely to occur (Verplanken et al., 2018). They have the potential to distance us from the contextual cues that trigger habitual responses automatically, leading to deliberate, informed actions becoming more likely (Verplanken and Aarts, 1999). While several studies have provided evidence in support of this account (e.g., Verplanken and Roy, 2016; Verplanken et al., 2018), important gaps remain in our understanding of moments of change, and the purpose of our study was to consider three such gaps. First, how the likelihood of behavioral change is affected by a composite intervention incorporating implementation intentions. To our knowledge, this is the first study to try to use implementation intentions to affect domestic water-use behavior. Here, we found that the intervention increased self-reported water-saving behavior but did not change shower time or residential water usage. Contrary to our expectation, we found evidence to suggest improvements in water conservation behavior irrespective of whether participants received the intervention or not. Second, we considered how differences in readiness to change and the strength of existing habits each affect the likelihood of change. Here, we found that the intervention was no more effective with greater readiness to change or with weaker existing habits. Third, we considered how the recency of the change event may make participants more or less susceptible to an intervention. Here, we found the intervention was no more effective for participants with a recent (1–2 months after starting at university) than an earlier change event (5–6 months after starting at university).

Our composite behavior-change intervention consisted of information on water conservation (cf. Whitmarsh et al., 2021), an implementation intention formation task (Bell et al., 2016), and performance feedback monitoring in the form of a shower-timer (e.g., Haggar et al., 2023). Control participants completed a reading comprehension filler task instead. Implementation intentions have been shown to be effective in changing habitual behavior (Gollwitzer and Oettingen, 2020) and so may be especially effective during moments of change, when habits are hypothesized to weaken (Verplanken et al., 2018). Additionally, composite interventions, in general, are thought to be more effective than interventions with only one element (Osbaldiston and Schott, 2012). We found evidence that *water-saving behavior* increased with the intervention (compared to the control) but shower-time *specifically*, and residential water-usage, did not differ between intervention and control conditions. This suggests that the intervention may have made participants more conscious of water-saving behaviors and more likely to engage in some of these. However, our findings are consistent with shower-time maintenance being an unpopular choice and/or difficult to implement in practice. Changing behavior to save water is only as effective in saving water as the behaviors that are changed and, on average, showering is accountable for the highest proportion of domestic water use, making up around 34% of domestic water usage (Waterwise, 2024). To the extent that showering behavior was not affected, our intervention may have had less overall influence upon overall water usage, and to the extent that it was the proportional effect will diminish shower volumes with decreasing shower time.

Stages of change theories of behavior change, such as the transtheoretical model (Prochaska and DiClemente, 1983), hypothesize that behavior change moves through discrete stages, each marking a progression toward behavior change. Hence, we hypothesized that participants with greater readiness to change (Bell et al., 2016), those in later stages, would be more likely to change than those with less readiness to change, those in earlier stages. While we found that, overall, those with higher readiness to change reported more water-saving behavior than those with lower readiness to change, this may reflect associations between readiness to change and behavior change motivations (e.g., pro-environmental orientation) and so may be explained as these individuals engaging in more water-saving from the outset. Yet, we found no evidence that readiness to change was related to water saving changes over the course of the experiment and/or with the intervention, implying that those with more readiness to change were as likely to increase their water saving during the study period as those with less readiness to change. This seems contrary to the way in which readiness-to-change has been found to affect implementation intention success (Bell et al., 2016; Armitage and Arden, 2008), so one implication is that the implementation intention element was not as effective as information and/or feedback elements. However, it is also worth considering that stages of change may be most appropriate to describe lengthy, effortful and deliberative process of behavioral change, and it is possible that water-saving behaviors showed rapid progress from contemplation to action, making readiness to change less relevant. Indeed, from a practical perspective, these findings seem to exclude the concern that first year university students may be unresponsive to the intervention due to being unready for change in their water-saving behaviors.

We hypothesized that the composite intervention would be more effective amongst participants with weaker water-use habits, because weaker habits may be most easily broken in comparison to strong habits, these being perhaps more generalized or ingrained (Verplanken and Aarts, 1999). We could not confirm this through our results. Likewise, we failed to confirm any difference in water use behavior between those with stronger and weaker habits, such as might be expected through the close relationship between habit strength and repeated behavior in context (Carden and Wood, 2018), namely that habits are learned and strengthened through repeated behavior, so using water habitually is likely the result of engaging in water-use behaviors, such as showering for longer. Having found no evidence to support our hypothesis that habits would moderate the efficacy of the intervention, one possibility is that the MoC, through habit discontinuity, weakened existing habits, making the strength of existing habit *less* important to the success of the intervention (Verplanken et al., 2018), but further investigation is warranted to show that this can be replicated.

While some researchers have suggested interventions as more effective within three months of a moment of change event (such as starting university), others consider enhanced efficacy up to 6-months after the event to be a possibility; hence we investigated interval periods of 1–2 month and 5–6 month between participants. We hypothesized that 1–2-month participants, for whom the MoC was more recent, would be more likely to respond to the water conservation intervention. We found no evidence to support this hypothesis, either for shower time changes or water-saving behavior changes. However, these analyses did involve evaluation of three-way interaction effects (between group (1–2 months or 5–6 months), intervention, and time) using relatively small samples, so it is possible that these results are type-II errors, particularly if the underlying effect is relatively weak, as has been indicated for habit discontinuity engendered sustainability (Verplanken and Roy, 2016). Another possibility is that the intervention is as effective at both timepoints and that it may be no less effective for an extended period, perhaps even up to 12-months (Thomas et al., 2016). Additionally, one of our findings suggests the presence of a boundary condition: shower time (irrespective of intervention) only showed reductions for those who participated 5–6 months after starting university, raising the possibility that 1–2 months may be too soon after the event itself, where the challenges of adjusting to a new environment may limit the possibilities for enacting behavioral change (Burningham and Venn, 2020). In the context of the academic year, participants at the start of the university, at 1–2 months, may have felt oversaturated with new information and activities, compared to the participants 5–6 months later, who were in the full swing of their academic studies. Therefore, differences in the structure of academic activities over time, or in how students responded to these differences, may be reflected in our results. However, it is also worth mentioning that (1) the groups participated at different times-of-year (October to December and February to April, respectively) and (2) the observed reduction was more consistent with an adjustment from a longer shower-time of 10 minutes to a normal shower-time of 8 minutes (cf. Haggar et al., 2023). Together, this raises the possibility that (over the winter) students began taking longer showers for their thermal comfort and are moving back to what is a normal shower-length for that time of year, rather than of making beneficial reductions to levels below the current normal.

Beyond the intervention, we found evidence that participants (irrespective of intervention or control participation) reduced their shower times, increased their water saving and reduced residential water usage. These findings are mutually supportive: it is less plausible that both subjective and objective measures would differ by chance, and the comparison of participants to non-participants (i.e., maximum residential occupancy) in the objective measure analysis raises the possibility that changes may be greater for *participants* compared to *non-participants*, and not (for example) merely a manifestation of contextual changes, such as public attitudes to water saving. Moreover, as our design assigned participants to condition at the residential level, this tends to exclude the possibility that random assignment might have been compromised through information-sharing or mutual use of shower-timers within residences. This leaves the possibility of the questionnaires functioning as an intervention. First, completing the questionnaires (containing questions about personal water usage) may have increased participant-awareness of these behaviors, prompting behavioral changes (cf. Wolfstenholme et al., 2020). This would tend to reflect informational and feedback components: water-saving behavior questions were symmetric with the hints/tips provided in the intervention and so may have conveyed similar information, and the act of reporting behavioral frequencies on days one and seven may have functioned as a minimal type of monitoring. Second, although participants were told during recruitment that the study aimed to explore the impacts of the cost of living on behavior change, they may have inferred that this study focused on water from the fact that half of the lifestyle questions and all of the habit questions were water-related. This may have caused demand characteristics, leading to reduced water usage to meet researcher expectations, which were inferred from the content of the questionnaires alone, or the manner of recruitment (Orne, 1962). This may account for the general trend in our findings whereby dependent variables showed improvement in both intervention and control groups. However, establishing effective controls in field research is also potentially limited with participants in similar social networks potentially discussed the study. These limitations, while problematic for research, are less problematic for practitioners, for whom general diffusion of water conservation is an objective. It is also important to reiterate that only intervention participants were exposed to practical water-saving information and asked to make if-then implementation plans.

This study had several important limitations. Theoretically, two recency conditions (1-2 months or 5-6 months following starting university) does not include a strong control group for testing the efficacy of starting university as an MoC or habit discontinuity, such as a comparison to a time-period before starting university, but this is not a salient limitation for studies testing the enhanced efficacy of concurrent interventions to change behavior (e.g., Verplanken and Roy, 2016). Practically, we encountered recruitment problems during the study, leading to relatively small sample sizes and prompting adjustments to recruitment and statistical analysis. Self-report measures of behavior are subject to biases, particularly to social desirability (Veseley and Klöckner, 2020). One way to allow for this is through collecting objective data for comparison. We made some use of available objective data, and this objective data was likely accurate (residence water use was closely correlated with the maximum occupancy of residences). However, our objective data measured the water use of larger buildings, rather than of individual students. Hence, each data-point was aggregated (across hundreds of individuals) and so does not capture the detail of changes in water-use of each individual, or of smaller groups such as flats or corridors within buildings. Hence, while each measurement may be quite accurate, this aggregation makes data less accurate in reflecting the changes in water use of individuals or smaller groups. However, when considered as data reflecting water-use within buildings, relatively few buildings were sampled (amounting to ten data-points), increasing the possibility that the relationship we found between participation in the study and building-level water use occurred by chance. Hence, a fruitful avenue for further work would be conceptual replication implementing additional measurements, such as by metering high water-usage devices in communal areas of residences (e.g., showers) to ensure data at a less aggregate level, albeit limited to water use from particular behaviors. A further limitation in our design was in assessing baseline levels of measured variables in the baseline questionnaire only after the intervention (or filler task) had been completed. This leaves open the possibility that the intervention task may have, to some extent, primed or otherwise influenced answers to later questions, confounding the effects of time and intervention. However, if this was an important biasing factor, then we'd expect group differences on measured variables at baseline, and no such differences were evident.

Theoretically, our results provide modest support for a MoC or habit discontinuity account of changes taking place in water saving when an intervention to change behavior is used shortly after students begin university. This is because we found only modest change in behavior attributable to the intervention and no clear differences attributable to the timing of the intervention. However, our findings are consistent with the idea that going to university may be initially, within 1–2 months, an *inopportune* moment to undertake such personal behavior changes (although further research is necessary to exclude the potential effects of seasonal variation in shower duration). This corresponds to the idea that some events, while they may involve changes in context and/or disruptions in existing habits that meet the criteria for moments of change, may, at the same time, also involve more immediate priorities, identity or role changes, lifestyle disruptions, or other demanding or stressful elements that tend to limit the extent to which a change is feasible rather than facilitate change (Burningham and Venn, 2020). This has been considered as a factor that might limit the effectiveness of interventions during moments of change, if the interventions are delivered during periods in the transition when people's attention is divided and they are correspondingly less receptive (Schäfer et al., 2012). Further research with respect to the context of life events could also offer insights into which events or stages (during moments of change) are disruptive of contextual cues while not being too stressful or demanding, thus clearing multiple barriers to successfully reforming unwanted behavioral habits.

For policy-makers concerned with achieving water use reductions, such as on a university campus, our results do not support a recommendation to intervene with students soon after their arrival, but to allow perhaps 5 months before attempting to raise awareness. While our results do not seem to support a thorough recommendation for use of the behavior change intervention techniques we employed (information, implementation intentions, and feedback), they were somewhat effective, and have proved effective techniques in changing different behaviors in the past (Osbaldiston and Schott, 2012). Hence, it remains an open possibility that these techniques may prove effective if they are enhanced or if some unknown impediment is overcome. In the present study, the intervention was text-based, and participants were trusted to put what they had read into practice for themselves, whereas greater involvement from the researcher team (e.g., through direct communication and monitoring) may provide social reinforcement. Psychologically, a commitment (e.g., a public declaration) can enhance adherence to a change attempt (Lokhorst et al., 2013) and to implementing the means to affect change, such as perhaps forming stronger implementation intentions at the outset. Also, factors that may have impeded the intervention exist. Perhaps the most important was motivation: while the information we provided was factual, with advice on reducing water use, such information may risk being too abstract, particularly given the meteorological climate of the UK, in which precipitation is abundant. While enhancing the intervention is one avenue, our results did raise the possibility that an elaborate intervention may be no less effective than merely asking participants to monitor their own actions as part of a scientific study. Unfortunately, such scientific monitoring may be intrusive when implemented at scale or beyond the ethical framework of research—it is possible that social marketing or awareness-raising campaigns may not inspire similar adherence. Hence, a comparable approach could be to collect and offer feedback digitally, such as by smart meter or smartphone application, to support long-term self-monitoring and open a channel for information provision and practical demonstration (Cominola et al., 2021).

## Data Availability

The raw data supporting the conclusions of this article will be made available by the authors, without undue reservation.

## Footnotes

^1^MoC varied systematically rather than being randomised across buildings because first-wave recruitment was initially targeted only at small buildings, due to these buildings containing water-meters for each set of 11–13 students living together as a single household. However, we faced difficulty in recruiting sufficient participants to make appropriate analyses and so decided, in wave 2, to broaden our recruitment to larger residences, where the potential for recruitment was greater, but water meter data was only available for buildings rather than for each household within the building.

^2^Data points were excluded if they were either over the third quartile+(3^*^Interquartile range) or under the first quartile-(3^*^Interquartile range). The outlying shower times were 45 min, 30 min, and 25 min.

^3^The final reading is slightly earlier due to the Christmas holidays, during which most students return to their family homes and, hence, water usage in residences is lower.

^4^This necessitates aligning and ranking data for each F test rather than each ANOVA.

## References

[B1] Aarts H. Dijksterhuis A. Midden C. (1999). To plan or not to plan? Goal achievement or interrupting the performance of mundane behaviors. Eur. J. Soc. Psychol., 29, 971–979.

[B2] Armitage C. J. Arden M. A. (2008). How useful are the stages of change for targeting interventions? Randomised test of a brief intervention to reduce smoking. Health Psychol. 27, 789–798. 10.1037/0278-6133.27.6.789 19025275

[B3] Arnett J. J. (2016). College students as emerging adults: the developmental implications of the college context. Emerg. Adulth. 4, 219–222. 10.1177/2167696815587422

[B4] Bamberg S. (2000). The promotion of new behavior by forming an implementation intention: results of a field experiment in the domain of travel mode choice. J. Appl. Soc. Psychol. 30, 1903–1922. 10.1111/j.1559-1816.2000.tb02474.x

[B5] Bell B. Toth N. Little L. Smith M. (2016). Planning to save the planet using an online intervention based on implementation intentions to change adolescent self-reported energy-saving behavior. Environ. Behav. 48, 1049–1072. 10.1177/0013916515583550

[B6] Beyers W. Goossens L. Vansant I. Moors E. (2003). A structural model of autonomy in middle and late adolescence: connectedness, separation, detachment, and agency. J. Youth Adolescence 32, 351–365. 10.1023/A:1024922031510

[B7] Blanca M. J. Arnau J. García-Castro F. J. Alarcón R. Bono R. (2023). Repeated measures ANOVA and adjusted F-tests when sphericity is violated: which procedure is best? Front. Psychol. 14:1192453. 10.3389/fpsyg.2023.1192453 37711324 PMC10499170

[B8] Bolton P. (2024). “Higher education student numbers,” in House of Commons Library. Available at: https://commonslibrary.parliament.uk/research-briefings/cbp-7857/ (accessed April 30, 2024).

[B9] Borman G. D. Grigg J. Rozek C. S. Hanselman P. Dewey N. A. (2018). Self-affirmation effects are produced by school context, student engagement with the intervention, and time: Lessons from a district-wide implementation. Psychol. Sci. 29, 1773–1784. 10.1177/0956797618784016 30183515

[B10] Burningham K. Venn S. (2020). Are lifecourse transitions opportunities for moving to more sustainable consumption? J. Consum. Cult. 20, 102–121. 10.1177/1469540517729010

[B11] Carden L. Wood W. (2018). Habit formation and change. Curr. Opin. Beh. Sci. 20, 117–122. 10.1016/j.cobeha.2017.12.009

[B12] Cominola A. Giuliani M. Castelletti A. Fraternali P. Gonzalez S. Herrero J.. (2021). Long-term water conservation is fostered by smart meter-based feedback and digital user engagement. NPJ Clean Water 4:29. 10.1038/s41545-021-00119-0

[B13] Crippa M. Oreggioni G. Guizzardi D. Muntean M. Schaaf E. Lo Vullo E.. (2020). Fossil CO2 and GHG Emissions of All World Countries – 2020 report, EUR 30358 EN. (Luxemburg: Publication Office of the European Union).

[B14] Dean A. J. Kneebone S. Tull F. Lauren N. Smith L. D. (2021). ‘Stickiness' of water-saving behaviours: what factors influence whether behaviours are maintained or given up? Resour. Conserv. Recy. 169:105531. 10.1016/j.resconrec.2021.105531

[B15] DEFRA (2023). Plan for Water: out Integrated Plan for Delivering Clean and Plentiful Water. Available at: https://www.gov.uk/government/publications/plan-for-water-our-integrated-plan-for-delivering-clean-and-plentiful-water/plan-for-water-our-integrated-plan-for-delivering-clean-and-plentiful-water (accessed April 30, 2024).

[B16] Drake E. C. Sladek M. R. Doane L. D. (2016). Daily cortisol activity, loneliness, and coping efficacy in late adolescence: a longitudinal study of the transition to college. Int. J. Behav. Dev. 40, 334–345. 10.1177/0165025415581914 28979055 PMC5624735

[B17] Elkin L. A. Kay M. Higgins J. J. Wobbrock J. O. (2021). “An aligned rank transform procedure for multifactor contrast tests,” in The 34th Annual ACM Symposium on User Interface Software and Technology (New York, NY: Association for Computing Machinery), 754−768. 10.1145/3472749

[B18] Environment Agency (2020). Meeting Our Future Water Needs: a National Framework for Water Resources. Available at: https://www.gov.uk/government/publications/meeting-our-future-water-needs-a-national-framework-for-water-resources (accessed April 30, 2024).

[B19] Erikson E. H. (1968). Identity: Youth and Crisis. New York, NY: WW Norton & Company.

[B20] Finley S. L. Basu N. B. (2020). Curbing the summer surge: permanent outdoor water use restrictions in humid and semiarid cities. Water Resour. Res. 56:e2019WR026466. 10.1029/2019WR026466

[B21] Fujii S. Garling T. (2005). “Temporary structural change: a strategy to break car-use habit and promote public transport,” in Traffic and Transport Psychology: Theory and Application, ed. G. Underwood (Oxford, UK: Elsevier), 585–592.

[B22] Garcia-Valiñas M. A. Athukorala W. Wilson C. Torgler B. Gifford R. (2014). Nondiscretionary residential water use: the impact of habits and water-efficient technologies. Aust. J. Agr. Resour. EC. 58, 185–204. 10.1111/1467-8489.12030

[B23] Gardner B. (2009). Modelling motivation and habit in stable travel mode contexts. Transport Res. F-Traf. 12, 68–76. 10.1016/j.trf.2008.08.001

[B24] Gardner B. Abraham C. Lally P. de Bruijn G. J. (2012). Towards parsimony in habit measurement: Testing the convergent and predictive validity of an automaticity subscale of the Self-Report Habit Index. Int. J. Behav. Nutr. Phy. 9, 1–12. 10.1186/1479-5868-9-102 22935297 PMC3552971

[B25] Gardner B. Lally P. (2018). “Modelling habit formation and its determinants,” in The Psychology of Habit: Theory, Mechanisms, Change, and Contexts, ed. B. Verplanken (Cham, Switzerland: Springer) 207–229.

[B26] Goette L. Leong C. Qian N. (2019). Motivating household water conservation: A field experiment in Singapore. PLoS ONE 14:e0211891. 10.1371/journal.pone.0211891 30893305 PMC6426227

[B27] Gollwitzer P. M. Oettingen G. (2020). “Implementation intentions” in Encyclopedia of Behavioral Medicine, eds M. Gellman and J. Turner (New York, NY: Springer), 1159–1164.

[B28] Gregory G. D. Leo M. D. (2003). Repeated behavior and environmental psychology: the role of personal involvement and habit formation in explaining water consumption. J. Appl. Soc. Psychol., 33:6, 1261–1296. 10.1111/j.1559-1816.2003.tb01949.x

[B29] Grilli G. Curtis J. (2021). Encouraging pro-environmental behaviours: a review of methods and approaches. Renew. Sust. Energ. Rev. 135:110039. 10.1016/j.rser.2020.110039

[B30] Hafner R. Fuertes A. Pahl S. Jones R. Gongolells M. Casals M. (2020). Results and insight gained from applying the EnergyCat energy-saving serious game in UK social housing. Int. J. Serious Games 7, 27–48. 10.17083/ijsg.v7i2.333

[B31] Haggar P. Whitmarsh L. Nash N. (2023). A drop in the ocean? Fostering water-saving behavior and spillover through information provision and feedback. Environ. Behav. 55, 520–548. 10.1177/00139165231201371

[B32] Hand M. Shove E. Southerton D. (2005). Explaining showering: a discussion of the material, conventional, and temporal dimensions of practice. Sociol. Res. Online 10, 101–113. 10.5153/sro.1100

[B33] Hayes L. Zinner L. Wise J. Carton J. (2019). Effects of a self-affirmation intervention on grades in middle school and first-year college students. J. Articl. Support Null Hypoth. 16:1.

[B34] He C. Liu Z. Wu J. Pan X. Fang Z. Li J.. (2021). Future global urban water scarcity and potential solutions. Nat. Commun. 12:4667. 10.1038/s41467-021-25026-3 34344898 PMC8333427

[B35] Holland R. W. Aarts H. Langendam D. (2006). Breaking and creating habits on the working floor: A field-experiment on the power of implementation intentions. J. Exp. Soc. Psychol. 42, 776–783. 10.1016/j.jesp.2005.11.006

[B36] IEA (2017). Water–Energy Nexus. Paris: IEA. Available at: https://www.iea.org/reports/water-energy-nexus (accessed April 30, 2024).

[B37] Lally P. Van Jaarsveld C. H. Potts H. W. Wardle J. (2010). How are habits formed: Modelling habit formation in the real world. Eur. J. Soc. Psychol. 40, 998–1009. 10.1002/ejsp.674

[B38] Locke E. A. Latham G. P. (2002). Building a practically useful theory of goal setting and task motivation: a 35-year odyssey. Am. Psychol. 57, 705–717. 10.1037/0003-066X.57.9.705 12237980

[B39] Lokhorst A. M. Werner C. Staats H. van Dijk E. Gale J. L. (2013). Commitment and behavior change: a meta-analysis and critical review of commitment-making strategies in environmental research. Environ. Behav. 45, 3–34. 10.1177/0013916511411477

[B40] Maréchal K. (2010). Not irrational but habitual: the importance of “behavioural lock-in” in energy consumption. Ecol. Econ. 69, 1104–1114. 10.1016/j.ecolecon.2009.12.004

[B41] Mishra P. Pandey C. M. Singh U. Gupta A. Sahu C. Keshri A. (2019). Descriptive statistics and normality tests for statistical data. Ann. Card. Anaesth. 22, 67–72. 10.4103/aca.ACA_157_18 30648682 PMC6350423

[B42] Mometrix (2023). Reading Comprehension Practice Test 2. Available at: https://www.testprepreview.com/modules/reading2.htm (accessed November 16, 2022).

[B43] Orne M. T. (1962). On the social psychology of the psychological experiment: with particular reference to demand characteristics and their implications. Am. Psychol. 17, 776–783. 10.1037/h0043424

[B44] Osbaldiston R. Schott J. P. (2012). Environmental sustainability and behavioral science: meta-analysis of proenvironmental behavior experiments. Environ. Behav. 44, 257–299. 10.1177/0013916511402673

[B45] Prochaska J. O. DiClemente C. C. (1983). Stages and processes of self-change of smoking: toward an integrative model of change. J. Consult. Clin. Psych., 51, 390–395. 10.1037/0022-006X.51.3.390 6863699

[B46] Ralph K. M. Brown A. E. (2019). The role of habit and residential location in travel behavior change programs, a field experiment. Transportation 46, 719–734. 10.1007/s11116-017-9842-7

[B47] Rebar A. L. Gardner B. Rhodes R. E. Verplanken B. (2018). “The measurement of habit” in The Psychology of Habit: Theory, Mechanisms, Change, and Contexts, ed. B. Verplanken (Cham, Switzerland: Springer), 31–49.

[B48] Rise J. Thompson M. Verplanken B. (2003). Measuring implementation intentions in the context of the theory of planned behavior. Scand. J. Psychol. 44, 87–95. 10.1111/1467-9450.00325 12778976

[B49] Schäfer M. Jaeger-Erben M. Bamberg S. (2012). Life events as windows of opportunity for changing towards sustainable consumption patterns? Results from an intervention study. J. Consum. Policy 35, 65–84. 10.1007/s10603-011-9181-6

[B50] Swaffield J. Whitmarsh L. Poortinga W. (2023). Interrupting the flow of water: Behavioural interventions and moments of change. Institute of Water J. 8, 4–5. Available at: https://orca.cardiff.ac.uk/id/eprint/166174/1/Swaffield_Main%20Text.docx.pdf

[B51] Thøgersen J. (2012). “The importance of timing for breaking commuters' car driving habits” in The Habits of Consumption, eds. A. Warde and D. Southerton (Helsinki: Helsinki Collegium for Advances Studies), 130–140. Available at: https://helda.helsinki.fi/collections/9a73282a-92eb-48a6-aa5a-33ed9a239e49 (accessed April 30, 2024).

[B52] Thomas G. O. Poortinga W. Sautkina E. (2016). Habit discontinuity, self-activation, and the diminishing influence of context change: evidence from the UK understanding society survey. PLoS ONE 11:e0153490. 10.1371/journal.pone.0153490 27120333 PMC4847906

[B53] Tijs M. S. Karremans J. C. Veling H. de Lange M. A. van Meegeren P. Lion R. (2017). Saving water to save the environment: contrasting the effectiveness of environmental and monetary appeals in a residential water saving intervention. Soc. Influence 12, 69–79. 10.1080/15534510.2017.1333967

[B54] Universities UK (2024). UUK/GuildHE Accommodation Code of Practice List of Registered Properties by Member: October 2023. Available at: https://www.universitiesuk.ac.uk/topics/students/student-support/accommodation-code-practice (accessed June 18, 2024).

[B55] Verplanken B. Aarts H. (1999). Habit, attitude, and planned behaviour: Is habit an empty construct or an interesting case of goal-directed automaticity? Eur. Rev. Soc. Psychol., 10, 101–134. 10.1080/1479277994300003529795394

[B56] Verplanken B. Orbell S. (2003). Reflections on past behavior: a self-report index of habit strength. J. Appl. Soc. Psychol. 33, 1313–1330. 10.1111/j.1559-1816.2003.tb01951.x

[B57] Verplanken B. Roy D. (2016). Empowering interventions to promote sustainable lifestyles: testing the habit discontinuity hypothesis in a field experiment. J. Environ. Psychol., 45, 127–134. 10.1016/j.jenvp.2015.11.008

[B58] Verplanken B. Roy D. Whitmarsh L. (2018). “Cracks in the wall: Habit discontinuities as vehicles for behaviour change” in The Psychology of Habit: Theory, Mechanisms, Change, and Contexts, ed. B. Verplanken (Cham, Switzerland: Springer), 189–205.

[B59] Verplanken B. Whitmarsh L. (2021). Habit and climate change. Curr. Opin. Beh. Sci. 42, 42–46. 10.1016/j.cobeha.2021.02.020

[B60] Veseley S. Klöckner C. (2020). Social desirability in environmental psychology research: three meta-analyses. Front. Psychol. 11, 1395. 10.3389/fpsyg.2020.01395 32793022 PMC7393925

[B61] Walton A. Hume M. (2011). Creating positive habits in water conservation: the case of the Queensland Water Commission and the Target 140 campaign. Int. J. Nonprofit Voluntary Sector Market. 16, 215–224. 10.1002/nvsm.421

[B62] Waterwise (2024). Save Water. Available at: https://www.waterwise.org.uk/save-water/ (accessed July 11, 2024).

[B63] Whitmarsh L. Poortinga W. Capstick S. (2021). Behaviour change to address climate change. Curr. Opin. Psychol. 42, 76–81. 10.1016/j.copsyc.2021.04.002 33991862

[B64] Wobbrock J. O. Findlater L. Gergle D. Higgins J. J. (2011). “The aligned rank transform for nonparametric factorial analyses using only anova procedures,” in Proceedings of the SIGCHI Conference on Human Factors in Computing Systems, 143–146.

[B65] Wolfstenholme E. Poortinga W. Whitmarsh L. (2020). Two birds, one stone: the effectiveness of health and environmental messages to reduce meat consumption and encourage pro-environmental behavioral spillover. Front. Psychol. 11, 577111. 10.3389/fpsyg.2020.577111 33117243 PMC7575709

