# Supplementary material for: Encouraging water-saving behavior during a “Moment of Change”: the efficacy of implementation intentions on water conservation during the transition to university
Source: Front Psychol. 2024 Nov 13;15:1465696. doi: 10.3389/fpsyg.2024.1465696 (PMC11598352; doi:10.3389/fpsyg.2024.1465696)
Supplement: Supplementary file 1 [file Table_1.DOCX]

**Supplement**

*This is a supplement to the article titled “Encouraging water-saving behaviour during a ‘Moment of Change’: the efficacy of implementation intentions on water conservation during the transition to university” published in* Frontiers in Psychology*. It contains the questionnaire materials used in this study. Some variations in the questionnaires between experimental conditions are noted, and notes are presented inside square-brackets.*

**Baseline Questionnaire**

INFORMED CONSENT TO PARTICIPATE IN THIS RESEARCH

This information sheet forms part of the process of informed consent. It should give you a basic idea of what the research is about and what your participation will involve. Please read this information sheet carefully and contact one of the researchers (details provided below) if you are not clear about any details of the project

1. *What is the purpose of the project?*

The project aims to explore engagement with different lifestyle behaviours and impacts of the cost of living

2. *Why have I been selected to take part?*

Anyone can take part, we are interested in hearing views from a wide range of people, so there are no selection requirements

3. *Do I have to take part?*

It is completely up to you to decide if you would like to participate. Before you decide to take part we ask you to carefully read this information, which will explain what the research is about and what your participation will involve. If you agree to take part, we will then ask you to select a box to verify you provide your consent to take part. However, if at any time you decide you no longer wish to take part in this project you are free to withdraw, without giving a reason.

4. *What will I be asked to do?*

You will be asked to complete 3 online questionnaires over a 21 day period, which will ask a series of questions relating to your perception of the cost of living, and your engagement with different lifestyle behaviours. These will be completed on day 1 (now), day 7, and then the final questionnaire on day 21. If you sign up you will be required to complete each of the 3 online surveys, in order to successfully complete the study.

5. *What are the exclusion criteria?*

There are no exclusion criteria.

6. *What are the possible benefits of taking part?*

You will be entered into a prize draw to win one of ten £50 Love2Shop voucher after completing the 3 surveys for this study. Please only sign up if you are happy to complete all 3 surveys. In addition, the information that you and other participants provide in this project will help us to understand how people engage with different lifestyle behaviours over time, and the impact that cost of living has upon this.

7. *What are the possible disadvantages and risks of taking part?*

There are no disadvantages or risks involved with taking part in the project.

8. *Will my participation involve any discomfort or embarrassment?*

The study will not involve any discomfort or embarrassment. All surveys will be completed online in private, and your answers will be treated confidentially, so nothing you say will be linked back to you.

9. *Who will have access to the information that I provide?*

Only the research team will have access to information that you provide. All records will be treated as confidential.

10. *What will happen to the data collected and results of the project?*

All data collected during the project including personal, identifiable data will be treated as confidential and kept in a locked cabinet in a locked room or on a secure file on the University of Bath’s secure server (X drive). This storage of data will be done in accordance with GDPR. Recorded data will not be kept for any longer than 5 years. Your name or other identifying information will not be disclosed in any presentation or publication of the research.

After the project has finished, we will also provide participants with a summary of the project results if they would like that. This summary will not include any identifiable information and will show the overall findings of the project.

11. *Who has reviewed the project?*

This project has been given a favourable opinion by the University of Bath, Psychology Research Ethics Committee (PREC) [reference: 23 016].

12. *How can I withdraw from the project?*

If you wish to stop participating before completing all parts of the project you can inform one of the below identified researchers by email. You can withdraw from the project at any time without providing a reason for doing so and without any repercussions.

If for any reason you wish to withdraw your data, please contact an identified researcher within two weeks of your participation. After this date it may not be possible to withdraw your data as some results may have been published or anonymized. Your individual results will not be identifiable in any way in any presentation or publication.

13. *University of Bath privacy notice*

The University of Bath privacy notice can be found here: <https://www.bath.ac.uk/corporate-information/university-of-bath-privacy-notice-for-research-participants/>.

14. *What happens if there is a problem?*

If you have a concern about any aspect of the project you should ask to speak to the researchers who will do their best to answer any questions. If they are unable to resolve your concern or you wish to make a complaint regarding the project, please contact the Chair of the Psychology Research Ethics Committee (PREC):

Dr Chris Ashwin

Email: [C.Ashwin@bath.ac.uk](mailto:C.Ashwin@bath.ac.uk)

Tel: +44 (0) 1225 383502

15. *If I require further information who should I contact and how?*

Thank you for expressing an interest in participating in this project. Please do not hesitate to get in touch with us if you would like some more information.

Name of Researchers: Freya Rennison; Kaloyan Mitev, Rebecca Hafner

Contact details of Researchers: fear20@bath.ac.uk;  kpm29@bath.ac.uk; rjh56@bath.ac.uk

PI: Lorraine Whitmarsh

Contact details of project PI: lw2253@bath.ac.uk

**In order to continue with the study we ask that you provide your consent by selecting the option below.**

1. I hereby fully and freely consent to my participation in this study
2. I do not consent to participate in this research

[Selecting Option 2 ended the questionnaire with thanks.]

**Please enter your student e-mail**, we need this information to contact you about Part 2 of the study, and enter you into the prize draw for the vouchers

E-mails will not be used for any other purpose, and your responses to the surveys will be identified by an anonymised participant number only, so nothing you say will be linked back to you.

|  |
| --- |

**How old are you?**

|  |
| --- |

**What is your gender?**

1. Male
2. Female
3. Non-binary / third gender
4. Prefer not to say

**Please select your accommodation from the list below** [this version asked to the 5-6-month group]

1. Polden Court
2. Wolfson
3. Marlborough Court
4. Solsbury Court
5. Acer (The Quads)
6. Chestnut (The Quads)
7. Damson (The Quads)
8. Ebony (The Quads)
9. John Woods Building
10. Osborne House

**Please select your number from the list below** [this version asked to the 1-2-month group, who were recruited from the same complex of numbered residences]

1

2

3

4

5

6

7

8

9

[… all integers in between 9 and 49 …]

49

50

51

Other

Could you please write down a personal identifier in the box below consisting of the first letter of your first name, followed by the month you were born, followed by the first letter of your last name; e.g. if your name is John Roberts and you were born in June your identifier will be J06R. This is necessary for identification during the follow-up studies.

|  |
| --- |

[The following intervention text and questions were given to those in the intervention conditions.]

Please read the following information which relates to water saving.

Firstly, why is water saving important? 97.5% of the world’s water is locked in seas and oceans, too salty for human use. And most of the remaining 2.5% is in the ice caps. So we humans depend on the tiny bit available as fresh water – an essential natural resource for life. But we don't just use water for drinking. We wash in it, clean with it, and use it to produce everything from clothing to food. Governments and water companies can make the biggest difference to the way that our freshwater is used, but reducing your own water footprint at home is a great way to make an impact as an individual. What can I do? Here is some information on some of the best ways you can try to save water at home. Please take some time to read through this carefully.

1. Brushing your teeth. Don't let your water consumption run out of control. Save 6 litres if water a minute by turning off the tap while you brush your teeth.

2. Shower with less. Every minute you spend in the shower uses between 12 – 17 litres of water. Set a timer on your phone to keep your showers short, sweet and water-saving. Aim to keep to 4 minutes or less. Ensure you only switch the shower on when you are ready to get in.

3. Reducing meat and dairy. Rearing animals for meat and dairy is incredibly water-intensive. It takes 2,500 gallons of water to produce only one pound of beef – the amount needed to make 4 hamburgers. If you eat one hamburger a week, that's 2,500 gallons of water a month, and 30,000 gallons of water in just one year. By cutting down on meat and dairy and aiming to have at least one vegetarian day a week you'll be helping to conserve water. You'll also be helping to protect the climate. The meat and dairy industries are big contributors to global warming.

4. Steam your veggies. Steam your food to cut water usage and retain more of the natural nutrients. If you do boil, try using the leftover water as a tasty stock for soups. Or let it cool and use it to water plants.

5. Washing up. Using a bowl to wash up rather than a running tap can help you to reduce your water and energy usage. In fact, you only need to run a typical tap for 95 seconds to fill up a washing up bowl.

6. Only boil the water you need. When making a cup of tea or coffee, only fill the kettle with the amount of water you need. You’ll save water and energy too.

7. Enjoy cold drinking water? Try keeping a jug of cold water in the fridge. Running the tap for cold water is a small everyday action that is really easy to change. Try making a habit of filling up a jug of water and putting it in the fridge every day so that you always have a supply of cold water without running the tap.

8. Washing food. Use a bowl in the sink when washing fruit or vegetables rather than continuously running the taps. You can then use the waste water to water your plants.

Now it's "Over to you!" Hopefully as you were reading the information above, you realised there might be a few behaviours that you could be doing to reduce your water use. If not, perhaps take a second to go back and have a look!

We would now like you to pick 4 of these behaviours to try over the next 7 days, and to make some plans for how, where, and when you will carry out these behaviours over the next 7 days. To help you make these plans, we have provided some boxes where you can enter some text to briefly describe the different parts of the plans. For the first text box, we would like you to tell us the situation in which you will try the behaviour. This will take the form of a "If/when" statement e.g., If I take a shower. For the second text box, we would like you tell us which of the energy saving behaviours you will do in that situation. This will take the form of a "I will" statement e.g., I will keep it to 4 minutes or less. Taken together, the text in the boxes will make your plan - "If I take a shower, I will keep it to 4 minutes or less". Please take a moment to have a look at the examples provided below and to have a think about which 4 water saving behaviours you have chosen to try, and the situations you will do them in. When you are ready, fill in the boxes below with your plans.

Example Behaviours

IF/WHEN --- I WILL

Example 1. If I am brushing my teeth --- I will turn the taps off

Example 2. When I take a shower --- I will keep it to 4 minutes or less

Example 3. When I get home today --- I will fill up a jug with water and put it in the fridge for chilled drinking water

Example 4. When I am washing up --- I will use a bowl of water rather than continuously running the taps

|  | IF/WHEN | I WILL |
| --- | --- | --- |
| Behaviour 1 | ❏ | ❏ |
| Behaviour 2 | ❏ | ❏ |
| Behaviour 3 | ❏ | ❏ |
| Behaviour 4 | ❏ | ❏ |

Please look at the list below, which relate to the water saving behaviours you have just chosen from. Please select which 4 behaviours you have chosen to target from the list.

1. Turning off taps when brushing teeth

2. Keeping shower time to 4 minutes or less

3. Reducing meat and dairy consumption and/or having at least one vegetarian/vegan day a week

4. Steaming veggies rather than boiling them

5. Washing up using a bowl of water rather than continuously running the taps

6. Only boiling the water you need when using the kettle

7. Keeping a jug of cold water in the fridge for drinking water

8. Using a bowl of water in the sink for washing vegetables rather than continuously running the taps

If you have chosen to target shower use as one of your 4 chosen behaviours, please consider using the shower timer provided to you at the start of the experiment when you take your showers over the coming weeks. This will help you to keep your shower time to 4 minutes or less, once you flip the timer it starts counting down 4 minutes. If you did not choose showering as one of your behaviours, you can still use the timer to track your shower time.

[The following intervention text and questions were given to those in the control conditions.]

Please read the following description of a group game that may be played in a later survey. Once you have read the passage please answer the questions underneath, which relate to how the game is played.

*One person starts off by naming anything that is geographical. It could be a city, state, country, river, lake, or any proper geographical term. For example, the person might say, “Boston.” The second person has 10 seconds to think of how the word ends and come up with another geographical term starting with that letter. The second participant might say, “Norway,” because the geographical term has to start with “N.” The third person would have to choose a word beginning with “Y.” If a player fails to think of a correct answer within the time limit, that player is out of the game. The last person to survive is the champion. This can be played in an online format, with typed responses.*

This game may help you with...

1. History
2. Music
3. Geography
4. Sports
5. Current events

The person trying to answer needs…

1. No time limit
2. To know geography only
3. To ignore the last letters of words
4. To know something about spelling and geography
5. To be a good speller

Before you choose your own word, think about how…

1. The last word starts
2. The last word ends
3. Smart you are
4. Long the last word is
5. The first word is spelled

The answer must be…

1. Within the United Kingdom
2. Within Europe
3. A proper geographical term
4. In the same region
5. Along a coast line

[All participants were asked all questions that follow. These questions were also repeated in questionnaires at 7-days and 21-days after baseline.]

**Thinking about the last 2 weeks, how often have you taken the following actions** (Please select one answer for each statement)

|  | Never | Very rarely | Sometimes | Often | Always |
| --- | --- | --- | --- | --- | --- |
| I have visited a historic, cultural landmark or other heritage location | ❏ | ❏ | ❏ | ❏ | ❏ |
| I have substituted one grocery product for another, e.g. semi-skimmed for full-fat milk | ❏ | ❏ | ❏ | ❏ | ❏ |
| I have eaten a meal with family or friends | ❏ | ❏ | ❏ | ❏ | ❏ |
| I have exercised or played sports | ❏ | ❏ | ❏ | ❏ | ❏ |
| I have cooked meals from scratch | ❏ | ❏ | ❏ | ❏ | ❏ |
| I have spent time in voluntary work e.g. befriending or answering a help-line | ❏ | ❏ | ❏ | ❏ | ❏ |
| I have spent time outdoors | ❏ | ❏ | ❏ | ❏ | ❏ |
| I have visited an outside place with water, such as a lake, pond, or the seaside | ❏ | ❏ | ❏ | ❏ | ❏ |
| I turn the tap off when I am brushing my teeth | ❏ | ❏ | ❏ | ❏ | ❏ |
| When boiling the kettle I have only filled it with as much water as a I need | ❏ | ❏ | ❏ | ❏ | ❏ |
| When cooking vegetables I have steamed them rather than boil when possible | ❏ | ❏ | ❏ | ❏ | ❏ |
| I have done my washing up using a bowl of water rather than continuously running the tap | ❏ | ❏ | ❏ | ❏ | ❏ |
| I have tried to keep my shower time to 4 minutes or less | ❏ | ❏ | ❏ | ❏ | ❏ |
| I have used a jug of water in the fridge for drinking water, rather than running the tap each time | ❏ | ❏ | ❏ | ❏ | ❏ |
| When washing fruit and vegetables I have used a bowl of cold water rather than continuously running the tap | ❏ | ❏ | ❏ | ❏ | ❏ |
| I have tried to minimise my meat and dairy consumption, and/or to have at least one vegetarian/vegan day a week | ❏ | ❏ | ❏ | ❏ | ❏ |

Thinking about the current “cost of living crisis”; (rising cost of home energy, petrol, food, and other goods).Please rate the extent to which this is affecting you at the moment from 1 (not at all) - 5 (a lot).

1. 1 (not at all)
2. 2
3. 3
4. 4
5. 5 (a lot)

**How long, on average do you spend in the shower?** Please write your answer in minutes in the box below. This can be a rough guess.

|  |
| --- |

Please identify the statement that most represents your current feelings towards your water use at home (select one option)

1. I currently do not try to save water and I am not thinking about starting
2. I currently do not try to save water but I am thinking about starting
3. I currently try to save water but not on a regular basis
4. I currently try to save water but have only begun to do so recently/in the last 6 months
5. I currently try to save water and I have done so for a long time/longer than 6 months

Please rate your agreement with the following statements from 1 (strongly disagree) - 5 (strongly agree)

|  | Strongly disagree  1 | Somewhat disagree  2 | Neither agree nor disagree  3 | Somewhat agree  4 | Strongly agree  5 |
| --- | --- | --- | --- | --- | --- |
| Using water in my home is something I do automatically | ❏ | ❏ | ❏ | ❏ | ❏ |
| Using water in my home is something I do without having to consciously remember | ❏ | ❏ | ❏ | ❏ | ❏ |
| Using water in my home is something I do without thinking | ❏ | ❏ | ❏ | ❏ | ❏ |
| Using water in my home is something I do before I realize I’m doing it | ❏ | ❏ | ❏ | ❏ | ❏ |
